# From functional neuroimaging to neurostimulation: fNIRS devices as cognitive enhancers

**DOI:** 10.3758/s13428-023-02144-y

**Published:** 2023-07-28

**Authors:** Jason Lee Waight, Natalia Arias, Ana M. Jiménez-García, Matteo Martini

**Affiliations:** 1 School of Psychology, University of East London, E15 4LZ London, UK; 2 Department of Basic and Clinical Neuroscience, Institute of Psychiatry, Psychology and Neuroscience, King’s College London, Denmark Hill, London, SE5 8AF UK; 3 Instituto de Neurociencias del Principado de Asturias (INEUROPA), 33005 Oviedo, Spain; 4 Health Research Institute of the Principality of Asturias (ISPA), 33011 Oviedo, Spain; 5 BRABE Group, Department of Psychology, Faculty of Life and Natural Sciences, University of Nebrija, C/del Hostal, 28248 Madrid, Spain; 6 Department of Humanities, Letters, Cultural Heritage and Educational Studies, via Arpi, 71121 Foggia, Italy

**Keywords:** fNIRS, PBM, Executive functions, Cognitive stimulation, Brain stimulation, Near-infrared light

## Abstract

Functional near-infrared spectroscopy (fNIRS) relies on near-infrared (NIR) light for changes in tissue oxygenation. For decades, this technique has been used in neuroscience to measure cortical activity. However, recent research suggests that NIR light directed to neural populations can modulate their activity through “photobiomodulation” (PBM). Yet, fNIRS is being used exclusively as a measurement tool. By adopting cognitive tests sensitive to prefrontal functioning, we show that a ‘classical’ fNIRS device, placed in correspondence of the prefrontal cortices of healthy participants, induces faster RTs and better accuracy in some of the indexes considered. A well-matched control group, wearing the same but inactive device, did not show any improvement. Hence, our findings indicate that the ‘standard’ use of fNIRS devices generates PBM impacting cognition. The neuromodulatory power intrinsic in that technique has been so far completely overlooked, and future studies will need to take this into account.

## Introduction

Functional near-infrared spectroscopy (fNIRS) is a neuroimaging technique that has gained increasing attention in recent years due to its safeness, transportability, good tolerance to movement-derived artifacts and practicality with hard-to-test populations (Lloyd-Fox et al., 2010). These characteristics endow fNIRS with greater ecological validity than traditional neuroimaging methods such as functional magnetic resonance imaging (fMRI) and positron emission tomography (PET) (Pinti et al., 2020). fNIRS measures changes in the brain's hemodynamic response, by using light spectroscopy at near-infrared (NIR) wavelengths. It operates on the principle that most biological tissues are semi-transparent to light in the NIR spectrum (600–1200 nm) (Henderson & Morries, 2015). However, when passing through blood vessels, a relatively high attenuation of NIR light occurs due to the presence of hemoglobin, which acts as a chromophore (Ferrari & Quaresima, 2012). Based on such mechanism, fNIRS has been traditionally used to detect temporal variations among the hemoglobin components, which can serve as a surrogate marker for neural activation (Arenth et al., 2007).

The main feature of the chromophores is their photon energy absorption, which constitutes the basis for the effects of “photobiomodulation” (PBM) (Hamblin, 2016). It has been suggested that PBM is based on photon energy absorption and upregulation of cytochrome c oxidase (CCO) (Wang et al., 2016, 2017), which is an enzyme found in the inner membrane of mitochondria that is essential for cellular metabolism (Arias et al., 2020; Méndez et al., 2021). NIR light interacts with CCO inside the mitochondria, restoring electron transport chain activity (Hamblin, 2018; Hennessy & Hamblin, 2017) and, therefore, improving energy metabolism. Indeed, there is evidence to suggest that sodium azide, a CCO inhibitor, moderates the impact of PBM (Spitler et al., 2015). Moreover, since CCO is an adaptive enzyme, the effects are long-lasting (Zomorrodi et al., 2019).

Recent research suggests that light in the NIR spectrum, projected into neural tissue, can also modulate neural activity and enhance cognitive functioning (Barrett & Gonzalez-Lima, 2013) through PBM. For instance, recent animal studies suggest that PBM applied shortly after a traumatic brain injury attenuates its impact (Shemesh et al., 2022) and there is an indication that PBM may improve the cognitive function of adults with traumatic brain injury (Naeser et al., 2011, 2014). Further evidence suggests that PBM may assist in managing the cognitive decline associated with ageing (Vargas et al., 2017), dementia (Nizamutdinov et al., 2021), and neurodegeneration (Arias et al., 2016; Méndez et al., 2021). Animal experiments suggest that PBM can improve spatial working memory in mice (Michalikova et al., 2008), and increase functional activity during the execution of a reversal task in rats (Gutiérrez-Menéndez et al., 2021).

A series of studies have also investigated whether PBM may improve cognitive function in healthy participants. In this regard, human studies have shown improvements in attention and short-term memory (Barrett & Gonzalez-Lima, 2013; Vargas et al, 2017), executive function (Chan et al., 2021), and rule-based category learning (Blanco et al., 2017). Similarly, it has been shown that PBM may enhance attentional capabilities during a Go/No-Go task (Jahan et al., 2019). Recently, the results of a study by Zhu et al. (2022) showed that besides a global cognitive function, PBM supported domain-specific effects improving attention, executive function, and working memory, while no amelioration was found in other subdomains of memory, like naming and visuospatial abilities. However, it is important to note that the three domains associated with PBM exist in a complex relationship. The alerting network, along with executive control and orienting, constitutes partially distinct networks supporting attention (Petersen & Posner, 2012). Therefore, the effects of PBM might be associated with changes in the alerting network, which supports achieving and keeping an alerting state (de Souza Almeida et al., 2021). Given that these three cognitive subdomains share common neuropsychological processes, it is plausible to observe simultaneous benefits due to these interventions after PBM. The underlying neural correlates of the effects of PBM might be related to the functional regulations on the frontoparietal regions, which are exactly the critical areas for the domains of attention, executive function, and working memory (Head et al., 1999; Andrés, 2003; Baldo & Dronkers, 2006; Scolari et al., 2015).

Also, regarding workload in cognitive tasks, animal studies have shown that when PBM was applied to groups of each sex for 5 days, no differences in brain metabolic activity or immediate early genes activation was observed compared to control groups (Gutiérrez-Menéndez et al., 2022). Those results are in contrast to the observed changes when PBM is applied under learning conditions. In this line, Gutiérrez-Menéndez et al. (2021) have explored the effect of PBM on experimental subjects with or without the execution of a learning task. Reversal memory was assessed using a Morris water maze and cytochrome c oxidase (CCO) was used as a brain metabolic activity marker. After 5 days of PBM the behavioral PBM group displayed CCO reduction in some regions involved in the execution of the reversal task, while the control PBM group showed a decrease of CCO levels in several brain regions. These results could show the effect of PBM on active brain networks and support the abovementioned effects of PBM on attention, executive functions and working memory.

Recently, it has been pointed out that despite the increasing evidence in favor of neuro-modulatory properties of NIR-light-based devices, fNIRS studies have not considered the potential effect derived from the use of their apparatus (Martini & Arias, 2021). Given fNIRS’ broad use in the neuroscientific field, it is essential to understand whether the adoption of such technique may have an effect on brain functionality. The results of the current research may shed light on the suitability of fNIRS in future investigations, especially when the aim is to validate/assess a treatment or an intervention through fNIRS.

Based on such premises, the present study seeks to test the potential modulatory effects on cognition deriving from the use of a traditional fNIRS device. In particular, we wanted to test whether the light stimulation via fNIRS translated into a better performance, with shorter reaction times and fewer errors made by the experimental group compared to the control group. A pseudo-randomized mixed-design was considered, with one between-participants factor (experimental and control group) and one within-participants factor (pre- and post-stimulation sessions). The inclusion of a control group accounted for mere learning effects. Participants undertook three tests of cognitive function (a delayed matched to sample task (DMS), a backwards counting task (BCT) and an eStroop test, before and while they were donning the fNIRS device. The device was applied in correspondence with the participants’ PFC, but only the experimental group had the device switched on. As previously described, it was hypothesized that the experimental group would have shown an improvement in their cognitive abilities. Specifically, we hypothesized that our experimental group would have shown better latencies and a higher number of correct trials in the DMS as in Barrett and Gonzalez-Lima’s study (2013) and a better performance at the eStroop color–word interference (incongruent) scores as in Martin and coworkers (2021) and Naeser et al. (2014). Since, to our knowledge, no PBM study has yet investigated the effects of transcranial light stimulation on the performance at the BCT, no specific hypotheses were formulated for this test.

## Materials and methods

### Participants

Thirty healthy English-speaking adult participants (18 males, 12 females) ranging from 19 to 51 years of age, with a mean of 35.0 years (SD = 10.9 years) were recruited for the study. Volunteer participants were recruited primarily through word of mouth. Inclusion criteria were: age between 18 and 55 and no history of head injury, psychiatric or neurological conditions. Participants were asked to confirm that they did not suffer from color blindness (to avoid confounding effects on the eStroop and DMS). A test for color blindness was available if participants were unsure (one potential participant withdrew following the test). The test takes 5 min and is available online (EnChroma® Color Blind Test | Test Your Color Vision).

Before the experiment, demographic information was collected to match the experimental and control groups’ age, gender, ethnicity, and years of education. Evidence suggests that cerebral hemispheric dominance (indicated by handedness) can impact cognitive performance. In particular, since handedness may determine an advantage on spatial tasks and psychophysics (Somers et al., 2015), participants were assessed for handedness using the Edinburgh Handedness Inventory (EHI, Veale, 2014) and counterbalanced across the control and experimental groups (each contained one left-handed participant).

Sleep deprivation may also impact cognitive performance (Killgore, 2010). Consequently, participants’ sleep duration from the night preceding the experiment was collected and used as a factor in determining allocation to either the control or experimental groups. Furthermore, as caffeine consumption has been shown to improve cognitive function in older adults (Nehlig, 2010), information on caffeine consumed on the day of the experiment was collected.

Finally, given that NIR light is absorbed by melanin (Wassenaar & Van den Brand, 2005), the ethnicity of our participants was balanced to have the same number of White, Asian, and mixed race between the two groups. Data relative to both groups are reported in Table 1. The first participant was allocated randomly. Subsequently, attempts were made to match participants' details with an existing participant with similar characteristics. The new participant was placed in the opposite group if a match was found. If no match was found, the participant was allocated randomly.

**Table 1:** Participant demographics

	All participants (*n* = 30)	Experimental group (*n* = 15)	Control group (*n* = 15)
Age/mean (SD)	35.0 (*10.9*)	34.9 (*11.0*)	35.2 (*11.1*)
Age range	19–51	20–51	19–51
Female gender (%)	40%	40%	40%
Years in education - mean (SD)	16.1 (3.0)	15.9 (3.0)	16.3 (3.1)
Ethnicity -White	24	12	12
Ethnicity - Asian	4	2	2
Ethnicity - Mixed	2	1	1
Coffee - no. of cups	0.9 (0.7)	0.9 (0.8)	0.9 (0.6)
Hours slept - mean (SD)	7.2 (1.0)	7.5 (0.9)	7.0 (1.0)
EHI - mean (No. left-handed)	81.7 (2)	78.3 (1)	85 .0 (1)

This study was conducted in accordance with the Declaration of Helsinki and approved by the local Ethics Committee.

### Questionnaires

A questionnaire was used to screen participants for color blindness, neurological conditions, and drug use. The same questionnaire also included questions on gender, age, ethnicity, and education to match the control and experimental groups.

The experiment used an online tool, the gorilla experiment builder (https://gorilla.sc). This was also used to randomize the order in which each participant undertook the tasks, and a record was maintained manually to ensure this was the case. The tool also delivered written instructions for each task and ensured a minimum break of 8 min between the two rounds of cognitive measures (pre- and post-stimulation sessions).

### Functional near-infrared spectroscopy (fNIRS)

A Spectratech OEG-16H (Spectratech Inc., Yokohama, Japan) was used for the experiment, with wavelengths of 840 and 770 nm, an output power of 5.0 mW/770nm, 5.0 mW/840 nm max. The device has six infrared light emission probes and a further six probes for infrared light detection, with a total of 16 channels for simultaneous measurement. However, in this case, no data on changes in cerebral blood flow were collected (only the experimental group had it turned on). The device is designed to be worn on the forehead as infrared light is strongly absorbed by hair and hair follicles. Consequently, the NIRS probes were placed on the forehead of the participants (Strangman et al., 2002). Care was taken when placing the device to make sure no hair was in between the forehead and the probes. All of the probes’ centers were set in a 15 × 3 cm matrix area. The center of the measurement unit was placed on the frontopolar (Fpz) region according to the international 10-20 system. As in similar studies using the same device, the measurement areas (in this case coinciding with the areas stimulated) cover Fp1, Fpz, Fp2, F7, and F8, and regions slightly lower than F3 and F4. Fp1 and Fp2 are located on the anterior pole of left and right frontal lobe, correspondent to the left and right inferior parts of the superior frontal gyrus, respectively. F7 and F8 are on the pars triangularis in the left and right inferior frontal gyrus, respectively, and F3 and F4 are on the left and right middle frontal gyrus, corresponding to the dorsolateral prefrontal cortex (DLPFC) (Kim et al., 2007; Okamoto et al., 2004).

### Cognitive tests

Three tests were used to assess a range of cognitive functions: BCT, DMS, and eStroop. Since order effects, such as those deriving from fatigue, may impact performance (Smith, 2017), the order of the three tests was randomized in both the initial baseline round and then again in the second fNIRS round.

The tasks were performed twice (pre- and post-stimulation sessions) by participants in a reasonable length of time (on average, the experiment lasted 1 h). Instructions for each task were displayed on the laptop screen, and participants were given the opportunity to ask any questions. Before each task, all participants received an online prompt to complete tasks as quickly and accurately as possible, and this prompt was also highlighted verbally by the researcher.

#### Backwards counting task

The DLPFC is activated by mental arithmetic or serial subtraction (Vansteensel et al., 2014). Counting backwards thus likely relies on neural substrates that near-infrared light delivered by fNIRS may impact. Participants were asked to count backwards verbally from 112 in sets of seven (e.g., 112, 105, 98…). The task was adapted from the "Serial Sevens" task and is considered a measure of concentration and information processing speed (Williams et al., 1996).

The number of errors and total time taken from when the participant said "112" and "zero" were recorded. In the event of an error, the experimenter provided the correct answer, and the participant continued from that number. The timing was undertaken using a stopwatch and recorded immediately after the test was over.

#### Delayed matched to sample task

The task measures information processing speed and has a short-term memory component (Barrett & Gonzalez-Lima, 2013). As with the other tasks chosen, the literature suggests it engages regions of the brain in the frontal lobes that PBM may impact, such as the frontoparietal and the DLPFC (Nieder & Miller, 2004) and a better performance at the DMS has been documented following PBM (Barrett & Gonzalez-Lima, 2013).

The DMS task in this experiment was based on the task used by Barrett & Gonzalez-Lima (2013). In line with that study, participants were given a short (less than 1 min) practice session before the first test was undertaken.

**Fig. 1:**
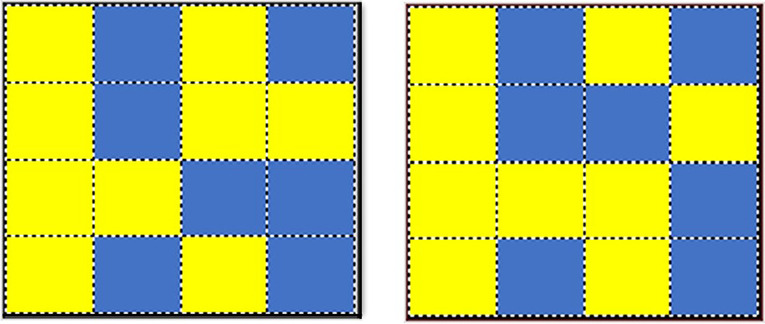
Example of stimuli used in the DMS task

During the task, participants viewed a 4 × 4 grid of blue (RGB value: 0 112 192) and yellow (RGB value: 255 255 0) squares (see Fig. 1). The grid consisted of between seven and nine blue squares, with the rest yellow. The stimuli were displayed for 5 s. Following this, the stimuli disappeared from the screen for 4 s. Two stimuli were then presented, one of which was the original stimulus. The other contained one or two switched squares. Participants were asked to click on the image seen previously using a mouse.

Whether the correct or incorrect stimuli were presented on the left or right of the screen was randomized, and a green tick or red cross informed participants whether the answer was correct or not. In line with Barrett and Gonzalez-Lima (2013), 30 trials lasting approximately 5 min were undertaken. The same block of 30 DMS trials was used for pre-and post-stimulation conditions. The program measured reaction time and accuracy. Participants were not given a time limit for studying the target or choosing the match but were asked to be as fast as possible while still trying to be accurate.

#### eStroop test

The Stroop test was chosen because it tests a range of executive functions and has previously been used to find evidence of enhanced cognitive function following PBM treatment (albeit in patients). For example, Naeser et al. (2014) used the test to assess improvements in the executive function of those with both mild and chronic brain injuries following the application of PBM.

The test was initially developed by Stroop (1935) and involves naming the colors in which color words are displayed. When those words conflict with the display color (e.g., the word “yellow” displayed in green), the participant is required to inhibit saying the word and instead must react to the color seen. The test has been used extensively to assess cognitive interference, but also working memory, attention, and processing speed (Scarpina & Tagini, 2017).

The experiment made use of eStroop, a custom-made script in Max 8 (Cycling ′74) developed by Brunetti et al. (2021), which offers a set of easily translatable stimuli capable of highlighting the main processes involved in the Stroop task and a proven voice key enabling the measurement of response times with millisecond precision.

The eStroop features four categories of stimuli in four different colors: (1) geometrical shapes, (2) neutral words (“two”, “three”, “four” and “seven”), (3) congruent color words (e.g., the word “red” displayed in red), and (4) incongruent color words (e.g., the word “blue” displayed in green). The four stimuli categories were presented in four different colors: red (RGB value: 255 0 0), blue (RGB value: 0 0 255), green (RGB value: 0 255 0) and yellow (RGB value: 255 255 0). All were presented on a grey (RGB value: 140 140 140) background (e.g., see “Fig. 1” in Brunetti et al., 2021). The eStroop was deployed on a laptop computer and used its microphone to record participants’ verbal responses.

During the test, participants were asked to name out loud the color of the stimuli presented. Hence, before running the real test, the experimenter performed a sound check to adjust the microphone's sensitivity to the participant's tone of voice. The eStroop tool is customizable but was used with the settings set out by Brunetti et al. (2021). Participants undertook 20 practice rounds prior to the first round of testing. Trials began with a fixation cross (of varying duration to avoid entrainment effects). In four blocks, 160 trials were performed (ten incidences of each color for all four conditions). Stimuli were presented randomly for at least 1500 ms or until the participant responded. The tool recorded participants' responses in digital sound files, and the researcher subsequently marked them for accuracy (using a module provided with the program). In line with Brunetti et al. (2021), corrections were marked as inaccurate. Response times were obtained and stored together with the results for accuracy.

### Procedure

Potential participants were given an information letter which set out the inclusion criteria. Upon arrival, participants were seated in a quiet room in front of a laptop computer. Lighting conditions and screen brightness of the laptop were identical for all participants and designed to maximize the visibility of the laptop screen and minimize the vision of the surrounding environment.

Participants were sat approximately 65 cm from a 13’ screen, on which the display of the consent form set the start of the experiment. A demographic’s questionnaire and the Edinburgh Handedness Inventory then followed. Participants were then assigned to either the control or experimental group following the process described above.

Following instructions and the opportunity to ask any questions, participants undertook the three tasks described above. The order of the tasks was randomized and each task started with its instructions. Only when the participant confirmed that the instructions were clear the task could start. On completing the first round of the three tasks, participants took a short break (around 2 mins) while the fNIRS device was fitted. In the case of the experimental group, the researcher ensured the device was switched on and that all 16 channels were operational through a calibration check (OEGSpO2.exe - Spectratech Inc., Yokohama, Japan).

**Fig. 2:**
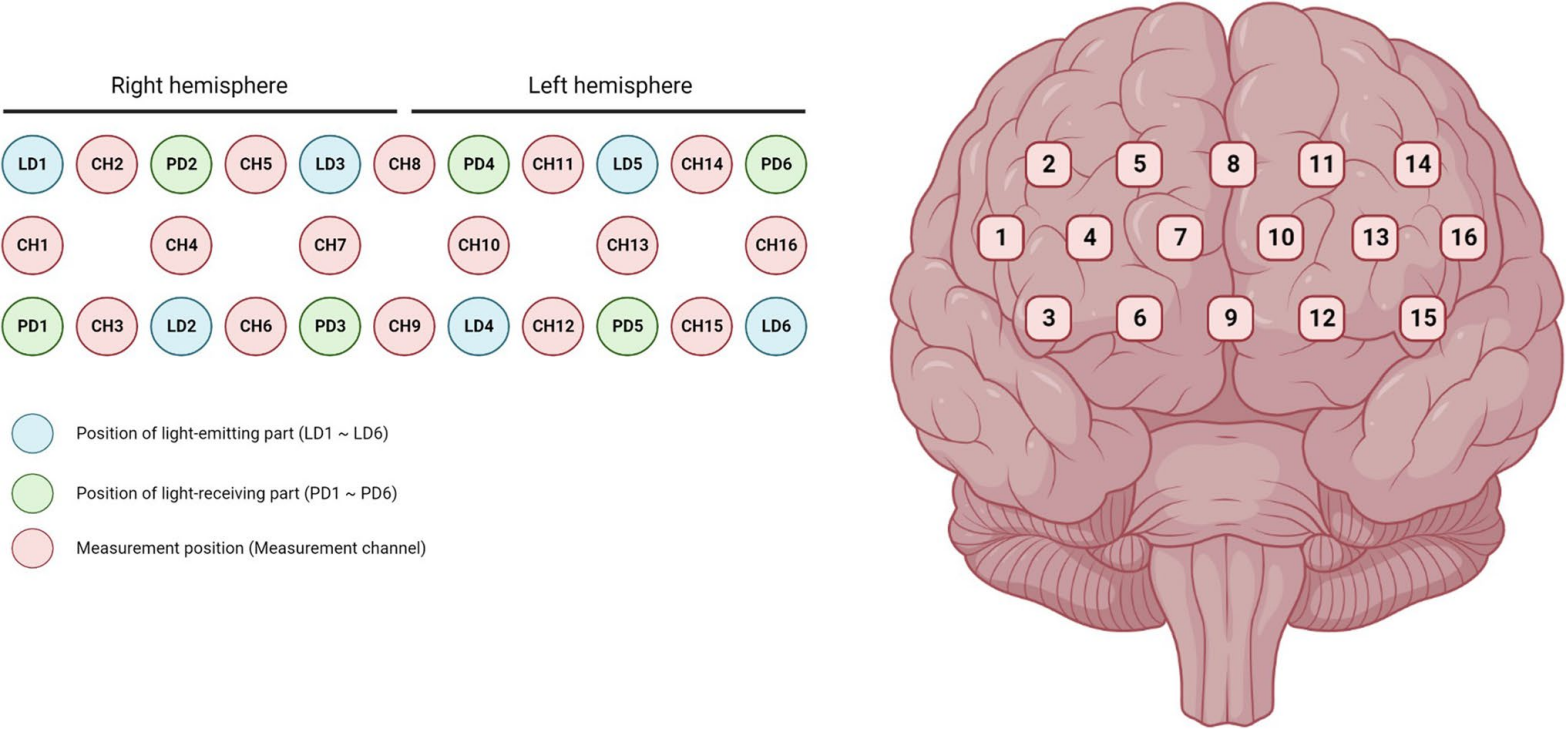
Spectratech OEG-16H Channels, light-emitting and light receiving parts positioning (*left*) and channels placement with respect to the cortex (*right*). Each measurement channel corresponds to a 2-cm-wide area in the brain irradiated by the NIR light

The center of the measurement unit was placed on the frontopolar (Fp) region according to the international 10-20 system, so that the array of channels was displayed in correspondence of the PFC (see Fig. 2).

Effective transcranial treatment times for PBM are typically 4–30 min (Carroll, 2019). In line with the treatment time used by Barrett and Gonzalez-Lima (2013), participants were therefore asked to focus on a fixation point on the laptop screen for 8 min following the fNIRS device being turned on. Participants in the control group were also asked to follow the same procedure with the device turned off.

The three cognitive tasks were then undertaken for a second time. The order of the tasks was again randomized. Participants wore the device for the duration of the second round of tasks (approximately 20 min). The device remained switched on throughout the “post” session for the experimental group. At the end of the third task, the fNIRS device was removed and participants debriefed.

### Design

This was a single-blinded cross-sectional 2 × 2 mixed ANOVA design with a within-factor ‘Session’, with two levels (‘pre’ session and ‘post’ session) and a between-factor ‘Group’, also with two levels (‘experimental’ and ‘control’).

### Data analysis

A 2 × 2 mixed ANOVA was performed for each of the cognitive tests evaluated. During the performance of a 2 × 2 mixed ANOVA, the omnibus test was carried out. If the interaction was significant, pairwise comparisons of the simple effects were considered.  When the interaction was not significant in the ANOVA, a main effects test was performed (omnibus test in unidirectional analysis of variance) conducting a post hoc analysis. This analysis is in accordance with Kirk (2013), who indicates that multiple comparison procedures from an ANOVA model should be carried out even if the interaction is not significant. Similar statistical approaches could be found in fNIRS studies (Han et al., 2022; Horiuchi et al., 2014).  In all statistical analyses performed, a confidence level of 95% and an alpha of 0.05 were considered to be statistically significant. Descriptive and inferential statistics were performed with SigmaStat, 3.5 version (Systat Software Inc., San Jose, CA, USA).

## Results

Prior to testing the results of the main variables, a between-group check in terms of age, sleep, coffee intake, and years of education as possible confounding factors (Branco et al., 2014; Buczylowska & Petermann, 2016; Killgore, 2010; Nehlig, 2010) was performed. As these variables were not normally distributed according to the Shapiro–Wilk (S-W) test (all *p*_s_ > 0.5) a Mann–Whitney test was adopted. The results of the comparisons per each variable did not show any significant difference between the two groups (age: *p* = 0.98; education years: *p* = 0.98; sleep: *p* = 0.19; coffee: *p* = 0.75).

**Fig. 3:**
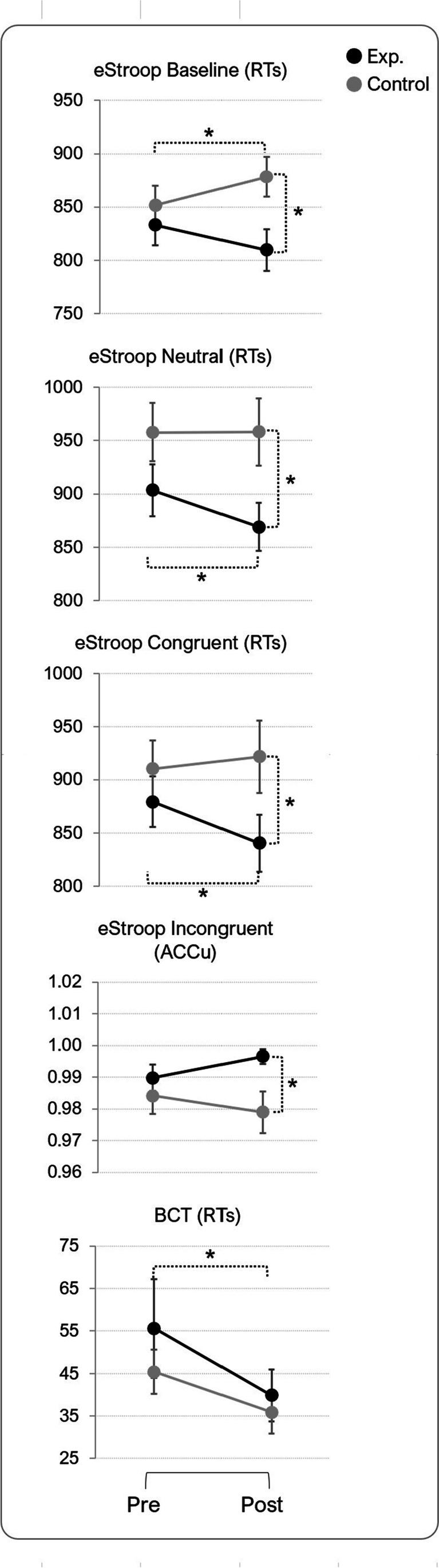
Plots showing the groups’ averages per each condition of the eStroop (first four plots from the top) and the BCT. *Error bars* are standard errors. Significant post hoc comparisons are reported with an *asterisk* (*p* < 0.05)

**Fig. 4:**
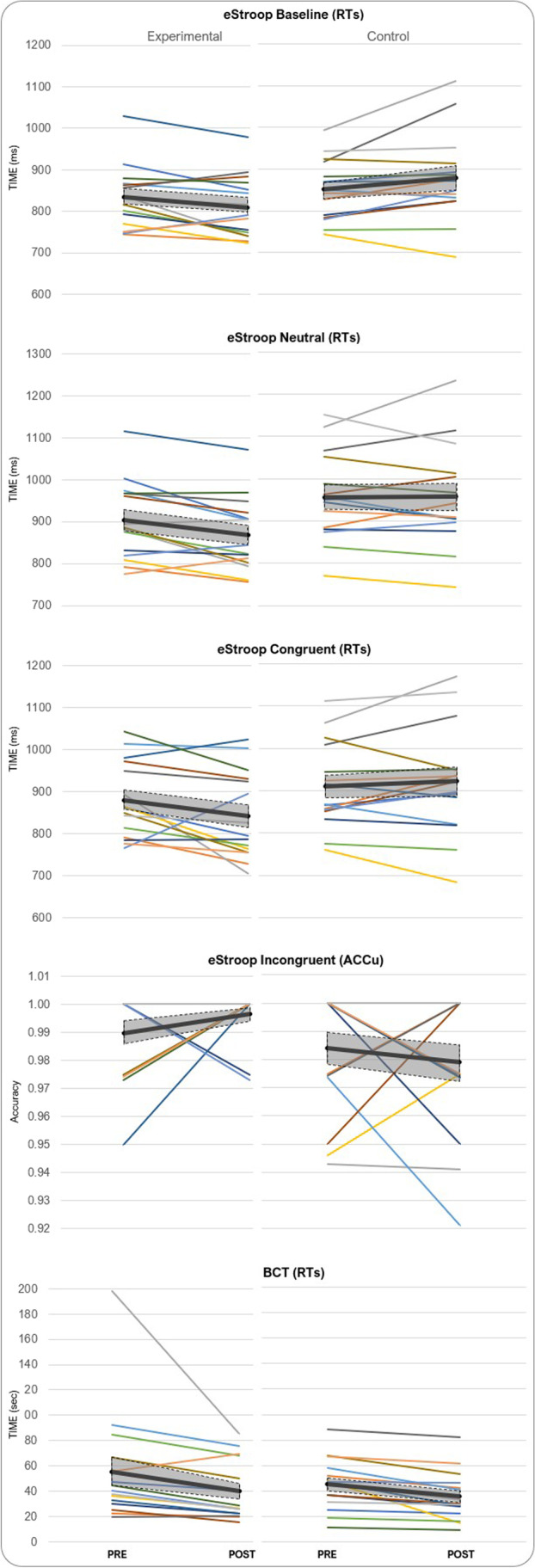
Spaghetti plots of the individual scores per each test that showed significant results, with group average (*line in bold*) and SE (grey area).

Subsequently, an analysis of the scores deriving from each cognitive test was performed. Response times and accuracy were assessed for (1) BCT, (2) DMS, (3) eStroop geometric shapes (discs) (eStroop-DSC baseline condition), (4) neutral words (eStroop-NEU), (5) eStroop congruent color words (eStroop-CNG) and (6) eStroop incongruent Color words (eStroop-INC). Response time and accuracy data were collected for all the pre- and post-sessions. Response times (RT) and accuracy (ACCu) scores from the cognitive tests, obtained as described below, are reported as group means in Table 2. Significant post hoc tests have been plotted and reported in Fig. 3, while individual scores are plotted in Fig. 4.

**Table 2:** Means and standard deviations for the cognitive measures

Measure		Group	Pre-stimulation		Post-stimulation		Change (%)
			Mean	SD	Mean	SD	
RT (s)	BCT	Exp	54.7	45.0	36.8	22.6	– 32.7*
		Con	46.3	20.2	39	20.5	– 15.8
	DMS	Exp	2.10	0.38	1.92	0.26	– 10.7*
		Con	2.19	0.57	2.14	0.72	– 4.6
	eStroop-DSC	Exp	0.83	0.07	0.81	0.08	– 3.6*
		Con	0.85	0.07	0.88	0.1	3.0
	eStroop -NEU	Exp	0.90	0.09	0.87	0.09	– 3.3*
		Con	0.96	0.11	0.96	0.12	0
	eStroop -CNG	Exp	0.88	0.09	0.84	0.10	– 4.6*
		Con	0.91	0.11	0.92	0.13	1.1
	eStroop -INC	Exp	0.97	0.10	0.93	0.10	– 3.1*
		Con	1.03	0.12	1.03	0.12	0
ACCu (%)	BCT	Exp	94.3	8.0	98.3	5.0	4
		Con	89.3	10.6	95.1	6.2	5.8
	DMS	Exp	91.2	6.8	91.1	6.6	0.8
		Con	91.8	5.8	93.5	6.8	2.6
	eStroop -DSC	Exp	99.9	0.5	99.9	0.5	– 0.2*
		Con	100	0	99.5	1.1	– 0.5
	eStroop -NEU	Exp	99.5	1.4	99.8	0.7	0.3
		Con	99.5	1.0	99.8	1.1	0.3
	eStroop -CNG	Exp	99.7	0.7	99.3	1.5	– 0.4
		Con	99.3	1.2	100	0	0.5
	eStroop -INC	Exp	99.0	1.6	99.7	0.9	0.9*
		Con	98.4	2.2	97.9	2.5	– 0.5

* *p ≤* .05 two-tailed

### BCT

For the BCT, two outcome measures were considered for each participant and session: (a) an Accuracy (ACCu) score, given by the sum of correct responses (each number correctly identified was counted as a correct response) divided by the total number of the responses (e.g., total numbers to be identified) and a (b) RT score, which corresponded to the total time taken to complete the whole counting (e.g., to reach ‘0’).

Since the variables obtained were not normally distributed according to the S-W test (all *p*s < 0.01) and some of them had negative skewness with values between – 1 and – 2 a logarithmic transformation was considered [Log10 (K-X), where K is = 1 + the highest number in the distribution (Barbaranelli, 2003)]. Despite such a transformation, the distribution did not improve, so we proceeded with the analysis of the original data. After all, the *F*-test has proven to be robust even to great departures from normality (Blanca et al., 2017).

#### ACCu

The mixed ANOVA revealed no effect of the factor ‘group’ (*F*_1,28_ = 1.03, *p* = 0.32, *η*^2^_p_ = 0.03). However, a significant main effect was registered for the factor ‘session’ (*F*_1,28_ = 10.24, *p* = 0.003, η^2^_p_ = 0.26) with, as expected, a better performance during the post session (M = 0.96, SD = 0.06) than during the pre session (M = 0.92, SD = 0.10). No significant interaction effect was found (*F*_1,28_ = 0.29, *p* = 0.60, *η*^2^_p_ = 0.00). Tukey post hoc tests did not reveal any significant comparison.

#### RTs

The mixed-measure ANOVA showed no effect of the factor ‘group’ (*F*_1,28_ = 0.03, *p* = 0.87, *η*^2^_p_ = 0.01). However, a significant main effect was registered for the factor ‘session’ (*F*_1,28_ = 11.70, *p* = 0.002, *η*^2^_p_ = 0.28) with, as expected, less time needed to complete the post session (in seconds: M = 37.2, SD = 21.2) than during the pre session (M = 50.27, SD = 34.53). No significant interaction effect was found (*F*_1,28_ = 2.23, *p* = 0.15, *η*^2^_p_ = 0.02). However, Tukey post hoc tests revealed that only the experimental group showed a significant reduction of RTs between the two sessions (*p* = 0.002).

### DMS

For each participant and session (pre-post) two outcome measures were considered: (a) an Accuracy (ACCu) score, given by the sum of correct responses divided by the total number of the responses and a (b) reaction time (RT) score obtained by the average of the RTs relative to the correct responses. Wrong responses were not considered, as they would have altered the reliability of the RTs (for instance by dragging down the average value with very fast-but-wrong responses). No other datum was discarded.

Normality was checked via a Shapiro–Wilk test (Ghasemi & Zahediasl, 2012). While the S-W test did not turn out to be significant for the RTs scores, the ACCu score was not normally distributed neither in its pre (*p* = 0.012) nor in post-session scores (*p* = 0.005). However, as the data for both ACCu scores had a limited skewness (pre = – 0.88, post = – .80) the deviation from normality was considered to be negligible (Barbaranelli, 2003).

#### ACCu

A 2 × 2 mixed-measure ANOVA with the between-factor ‘group’ (experimental and control) and the within-factor ‘session’ (pre and post) was considered to analyze the ACCu scores of the DMTS. The results from the ANOVA showed no main effect of the factor ‘group’ nor of the factor ‘session’ (*F*_1,28_ = 0.60, *p* = 0.44, *η*^2^_p_ = 0.02 and *F*_1,28_ = 0.27, *p* = 0.61, *η*^2^_p_ = 0.01, respectively). The interaction effect was also not significant (*F*_1,28_ = 0.40 *p* = 0.54, *η*^2^_p_=0.01).

#### RTs

As for the ACCu score, a 2 × 2 mixed-measure ANOVA was run to analyze the RT scores. No main effect of the factor ‘group’ nor of the factor ‘session’ was found (*F*_1,28_ = 0.79, *p* = 0.38, *η*^2^_p_ = 0.02 and *F*_1,28_ = 2.77, *p* = 0.11, *η*^2^_p_ = 0.09, respectively). The interaction effect was also not significant (*F*_1,28_ = 0.92, *p* = 0.35, *η*^2^_p_ = 0.03).

### eStroop

As for the DMS task, two outcome measures were considered for the data deriving from the eStroop: an ACCu score and a RT, both obtained as reported above. Again, besides wrong responses, no other datum was excluded. The calculation of both scores was obtained for all four categories of stimuli, e.g., ‘geometrical shapes’ (baseline condition), ‘neutral’, ‘congruent’, and ‘incongruent’ words.

Normality was tested with a Shapiro–Wilk test. While the S-W test did not turn to be significant for the RTs scores (all *p*_s_ > 0.05), the ACCu score was not normally distributed neither in its pre nor in its post session scores, for any category (all *p*_*s*_ < 0.001). Contrarily to what was true for the ACCu scores of the DMS, here the big majority of the data reported a marked negative skewness (range – 5.47 to – 1.20). Due to scores close to ceiling effects in all conditions and sessions, transformations were not effective, thus, the results of the 2 × 2 mixed ANOVA on the accuracy scores need to be taken with caution. Nonetheless, as previously described, the *F*-test has proven to be robust to great departures from normality (Blanca et al., 2017).

#### ACCu

A 2 × 2 mixed-measure ANOVA with the between-factor ‘group’ (experimental and control) and the within-factor ‘session’ (pre and post) was considered to analyze the ACCu scores for each condition.

For the baseline condition, no main effect of the factor ‘group’ nor of the factor ‘session’ was found (*F*_1,28_ = 0.25, *p* = 0.62, *η*^2^_p_ = 0.02 and *F*_1,28_ = 1.94, *p* = 0.18, *η*^2^_p_ = 0.07, respectively). The interaction effect was also not significant (*F*_1,28_ = 1.94, *p* = 0.18, *η*^2^_p_ = 0.07).

For the neutral condition, no main effect of the factor ‘group’ nor of the factor ‘session’ was found (*F*_1,28_ = 0.12, *p* = 0.73, *η*^2^_p_ = 0.002 and F_1,28_ = 0.88, *p* = 0.36, *η*^2^_p_ = 0.009, respectively). The interaction effect was also not significant (*F*_1,28_ = 0.13, *p* = 0.72, *η*^2^_p_ = 0.002).

For the congruent condition, no main effect of the factor ‘group’ nor of the factor ‘session’ was found (*F*_1,28_ = 1.13, *p* = 0.73, *η*^2^_p_ = 0.005 and *F*_1,28_ = 0.08, *p* = 0.78, *η*^2^_p_ = 0.005, respectively). However, the interaction effect was found to be significant (*F*_1,28_ = 4.92, *p* = 0.04, *η*^2^_p_ = 0.13). Nonetheless, subsequent Tukey post hoc tests did not disclose any significant contrast (all *p*_s_ > 0.05).

For the incongruent condition, a main effect of the factor ‘group’ was found (*F*_1,28_ = 4.97, *p* = 0.03, *η*^2^_p_ = 0.15) with the experimental group doing overall better than the control group (M = 0.99, SD = 0.01 vs. M = 0.98, SD = 0.02). No main effect of the factor ‘session’ nor any significant interaction effect were evidenced (*F*_1,28_ = 0.03, *p* = 0.87, *η*^2^_p_ = 0.07, and *F*_1,28_ = 1.65, *p* = 0.21, *η*^2^_p_ = 0.07, respectively). However, Tukey post hoc tests revealed a significant difference in the post session between the two groups (*p* = 0.015), with the experimental group reporting a better performance.

#### RTs

As for the ACCu scores, a 2 × 2 mixed-measure ANOVA with the between-factor ‘group’ and the within-factor ‘session’ was considered to analyze the RT scores for each one of the four conditions.

Taking into account the data gathered in the baseline condition, no main effect of the factor ‘group’ nor of the factor ‘session’ was found (*F*_1,28_ = 2.23, *p* = 0.15, *η*^2^_p_ = 0.07 and *F*_1,28_ = 0.04, *p* = 0.84, *η*^2^_p_ = 0.001, respectively). However, the interaction effect was found to be significant (*F*_1,28_ = 8.68, *p* = 0.006, *η*^2^_p_ = 0.24). Subsequent Tukey post hoc tests showed differences between experimental groups within the post session where the experimental group performed faster than control (*p* = 0.03). Also, differences were found within the control group’s pre- and post-sessions with a significant worsening of the performance in the post session (*p* = 0.035).

For what concerns the neutral condition a trend towards significance for the factor ‘group’ was revealed (*F*_1,28_ = 3.95, *p* = 0.057, *η*^2^_p_ = 0.12). A significant main effect of the factor ‘session’ was disclosed (*F*_1,28_ = 4.61, *p* = 0.04, *η*^2^_p_ = 0.14) with, as expected, faster RTs during the post session (M = 913.6, SD = 113.2) than during the pre session (M = 930.7, SD = 101.6). The interaction effect was also significant (*F*_1,28_ = 4.71, *p* = 0.039, *η*^2^_p_ = 0.14). Tukey post hoc tests disclosed a significant difference in the RTs of the experimental group, with the RTs from the post session being significantly reduced compared to those measured during the pre-session (pre: M = 903.5, SD = 90.3, vs. post: M = 869.1, SD = 84.1, *p* = 0.027). Importantly, the control group did not show any reduction of its RTs in the post session compared to its pre session (pre: M = 957.9, SD = 105.1, vs. post: M = 958.1, SD = 121.4). Furthermore, a significant difference between the post session of the two groups was also highlighted (*p* = 0.022).

For the congruent condition, no main effect of the factor ‘group’ nor of the factor ‘session’ was found (*F*_1,28_ = 2.20, *p* = 0.15, *η*^2^_p_ = 0.07 and *F*_1,28_ = 1.38, *p* = 0.25, *η*^2^_p_ = 0.05, respectively). However, the interaction effect was found to be significant (*F*_1,28_ = 4.64, *p* = 0.04, *η*^2^_p_ = 0.14). Tukey post hoc tests disclose significant differences in the RTs of the experimental group, with the RTs from the post session being significantly reduced compared to those measured during pre session (pre: M = 879.6, SD = 91.0, vs. post: M = 840.6, SD = 103.8; *p* = 0.026). Also, significant differences were found in the post session between control and experimental groups, showing faster RTs for the experimental than for the control group (exp: M = 840.6, SD = 103.8, vs. control: M = 921.75, SD = 130.7; *p* = 0.048), which did not show any reduction in the RTs in the post session comparted to pre.

For the incongruent condition, trends towards significance both for the factor ‘group’ and ‘session’ were found (*F*_1,28_ = 3.85, *p* = 0.060, *η*^2^_p_ = 0.12 and *F*_1,28_ = 3.79, *p* = 0.062, *η*^2^_p_ = 0.12, respectively). Another trend towards significance was found when analyzing the interaction between the two factors (*F*_1,28_ = 3.32, *p* = 0.079, *η*^2^_p_ = 0.10). Although the comparisons were not significant, it is clear that the trends were driven by the experimental group (pre: M = 969.3, SD = 98.3; post: M = 932.9, SD = 98.8) doing overall better than the control group (pre: M = 1026.3, SD = 115.1; post: M = 1025.1, SD = 116.1).

## Discussion

The present study was the first aimed at evaluating the impact of near-infrared light, delivered via a fNIRS device, on cognition. As hypothesized, our results show that by merely turning on a device classically used for fNIRS, a modulation in the cognitive performance of healthy adults can be witnessed.

The improvement in performance found in the current research was unveiled by better RTs in the post session of the BCT and in the post session of almost all eStroop conditions. The overall accuracy at the incongruent condition of the eStroop also revealed a significant improvement for the experimental group as hypothesized. While recent research has shown the potential benefits of PBM on frontal cognitive functions (e.g., Jahan et al., 2019, Chan et al., 2021), this is the first study to show that a ‘classical’ fNIRS device used to measure brain activity could in fact be used as a cognitive stimulator, too. Indeed, in the first three conditions of the eStroop, the relatively short NIR light stimulation received by the experimental group brought about a significant increase in the speed these participants responded. Such improvement was present in the fourth condition (incongruent) as well as in the form of accuracy. The better accuracy reported by the experimental group was coupled with a decrease of RTs during this condition, while the incongruent condition’s RTs for controls practically showed no change at all. At last, contrarily to what was hypothesized, no statistically significant improvement was recorded in the DMS of the experimental group. Our findings are discussed below per each test considered in the present study.

### eStroop

Whether the performance in the Stroop task and the resulting Stroop effect involve higher-order cognitive level processes of control (conflict monitoring) or, more simply, input-driven selective attention, is still a matter of debate (Algom & Chajut, 2019). However, the incongruent condition is still renowned to be the most challenging with respect to the other conditions, having typically longer RTs compared to the others (MacLeod, 1991). This could explain why, in our study, the effect of the stimulation on the performance was less clear during the incongruent condition, at least for what concerned the RTs. fMRI studies (e.g., Blasi et al., 2006) consistently suggested an activation of the DLPFC in relation to tasks (including the Stroop test) that require cognitive control. It is known that the prefrontal region plays a significant role during both congruent and incongruent trials of the Stroop task (Jalalvandi et al., 2020). Given its location in the frontal lobes, the DLPFC might be the key neural region that our NIR light stimulation could stimulate, and its involvement may be responsible for the better performance at the eStroop task. Previous studies using the same task and similar fNIRS devices have reported a strong bilateral activation of the DLPFC especially during the incongruent trials (Schroeter et al., 2002). Other regions have also been identified as playing a role in conflict management and attentional control, such as the ACC.

Comparing the effects of the stimulation used in our experiment on the Stroop task with findings from other studies might prove challenging, as there are no current examples in the PBM literature of the Stroop task being used to test enhanced cognitive functions in healthy adults. However, research on participants with traumatic brain injury (TBI) supports the finding in this experiment that PBM can enhance executive function as measured by the Stroop test. Both a case study on two participants with TBI (Naeser et al., 2011) and a pilot study of 11 participants with chronic TBI (Naeser et al., 2014), suggest that repeated PBM treatment can improve executive function as measured by the Stroop test. However, no improvement in Stroop test performance was found following PBM treatment on participants with Gulf War Illness (Martin et al., 2021). Yet, its authors considered that the number of cognitive measures deployed when compared to the number of participants likely meant that this study was underpowered.

### BCT

Our results also show that only the experimental group reported a significant reduction of RTs between the two sessions of the BCT. fMRI studies on brain activation during mental arithmetic operations suggest the involvement of both prefrontal and parietal regions, with the bilateral intraparietal sulcus providing basic quantity representation and manipulation and prefrontal areas controlling the management of successive operations in working memory (Dehaene et al., 2004). Moreover, EEG-based research, specifically on counting backwards tasks, suggests that the ACC and left inferior parietal lobe are deployed during the task, but also noted the involvement of the medial prefrontal cortex (mPFC) (Kitaura et al., 2017). Similarly, an fMRI study on the task also suggests an important role for the posterior parietal area, but with consistent activation of the PFC (Rueckert et al., 1996). It is therefore plausible that, given the involvement of the PFC in BCT tasks, the NIR light in this experiment produced faster response times compared to those in the experimental group.

### DMS

In relation to the delayed match-to-sample test (DMS), the literature suggests that during this task a neurofunctional network is engaged which, among other brain areas, comprehend the right DLPFC (Daniel et al., 2016). Consistent with this finding, PBM treatments that have targeted the right side of the forehead have been shown to modulate performance on a DMS task (Barrett & Gonzalez-Lima, 2013; Vargas et al., 2017). In the present study, the improvement in response times from the experimental group in the DMS task (10.7%) was more than twice that of the control group (4.6%). However, neither the accuracy nor response times results achieved statistical significance, in contrast to two comparable studies that used the same task and a single PBM treatment (Barrett & Gonzalez-Lima, 2013). A potential explanation for such inconsistency may lie in the fact that this study had a smaller number of participants (30 compared to 40 and 60, respectively) so may have lacked sufficient power to detect an effect. Furthermore, both previous studies found that DMS accuracy declined in the control group in the post stimulation session, while our control group showed an improvement. As in the aforementioned studies, the DMS was always the last condition. This suggests PBM may have a protective effect against fatigue (Barret & Gonzalez-Lima, 2013). However, in the present experiment, the order of the tests was counterbalanced, so the DMS task may have been undertaken at the beginning, middle, or end of the last session. This protective effect may have been spread across the three cognitive measures, potentially making it harder to detect. It may also be that rather than enhancing performance, PBM makes cognitive tasks less demanding in terms of neural resources. A significant reduction in frontal hemodynamic levels following PBM treatment has been identified during an n-back task which, like the DMS, tests working memory, suggesting that PBM could make the task less effortful when high memory loads are involved (Chan et al., 2021).

#### Task differences

Despite the fact that the present experiment was designed to assess the prefrontal lobe abilities, we did not find significant results for the scores of all the tests we used. Since they measure cognitive abilities subserved by the same brain area, it would be plausible to assume a strong correlation among the scores of these tests. So, an intervention thought to manipulate the functionality of the neurobiological structure they reflect the activity of, should lead to an ‘equally’ visible variation in all of them. However, it should be noted that not only different factors may intervene in shaping the results (some of which have been described above), but also that many of these tests may in fact not be significantly correlated (Boone et al., 1998; Testa et al., 2012). For instance, tasks which are supposed to reflect ‘cognitive control’ should be differentiated between those measuring “proactive” control from those measuring “reactive” control processes (Enriquez-Geppert et al., 2014). The distinction between “proactive” and “reactive” finds a physiological basis in the different topographical distribution of theta oscillations, linked to cognitive control (Senoussi et al., 2022). Mainly proactive control-recruiting tasks like the DMTS would show a focal frontal distribution, whereas for primarily reactive control-recruiting tasks like the Stroop, the topographical distribution of theta oscillations would be much broader (Eschmann et al., 2018). Also, the Stroop test, which has traditionally been considered a tool for assessing the ability to inhibit cognitive interference (Scarpina & Tagini, 2017), according to a factor analysis study, may be more sensitive in gauging variations in information processing speed, ‘loading’ within a separate factor, compared to other executive functions tasks (Boone et al., 1998). Hence, it is no surprise that the same intervention (in our case the induction of PBM through fNIRS) may affect distinct aspects of the executive functioning, as measured by our distinct tests, in a different manner.

#### PBM and fNIRS

Near-infrared light application at specific wavelengths has potentially remarkable protective abilities against mitochondrial dysfunction and neuronal cell death in models of stroke, Parkinson’s disease, Alzheimer’s disease, and TBI (e.g., Berman et al., 2017; Lee et al., 2017). However, a few studies have proved that combined wavelengths could have a superior outcome. Mendez et al. (2003) compared, histologically, the effect of using two different wavelengths (GaAlAs 830 nm and InGaAl 685 nm). They concluded that better results were observed when combining both wavelengths of 830 and 685 nm and attributed this advantage to different absorption and penetration. Also, Zare et al. (2019) explored the influence of combined and/or single applications of red and near-infrared PBM at different wavelengths on mesenchymal stem cells and human adipose-stem cells. The results showed that PBM with the combined 630 + 810-nm laser significantly stimulated cell viability and significantly decreased apoptosis of both cell types in vitro. The irradiation parameters used in this study were different when compared to those that have previously identified a statistically significant effect (Barrett & Gonzalez-Lima, 2013; Blanco et al., 2017; Vargas et al., 2017). Those studies have used similar parameters but a single wavelength: the laser beam area measured 13.6 cm^2^, irradiation time was 8 min, and the power output was 3.4 W. The irradiance (power density) used was 250 mW/cm^2^, the cumulative fluence (energy density) 60 J/cm^2^ and the wavelength used was 1064 nm. By comparison, the Spectratech OEG-16H combined two wavelengths in the near-infrared spectrum with a beam area of 7.54 cm^2^ (6 LEDs with a 4-mm diameter) and emits 5 mW of power, which provided a total energy density of 2.4 J/cm^2^. These data support the potential therapeutic role of near-infrared light delivery via fNIRS devices by combining two different wavelengths targeting mitochondria among other biological chromophores involved in cognition.

To better understand the beneficial effects of photobiomodulation (PBM) on target tissues, it is important to note that similar outcomes can be achieved using different devices and wavelengths (800, 1000, or 1200 nm) by adjusting light stimulation duration or light emission power. This standardization of treatment across the field is crucial for investigators with different approaches to achieve consistent and quantifiable results (Pitzschke et al., 2015; Khan & Arany, 2016). It is also worth noting that near-infrared light could increase the release of nitric oxide (NO), which is responsible for the increased cerebral blood flow (Lee et al., 2017). NO is a major neuronal signaling molecule which, among other functions, possesses the ability to trigger vasodilation. This vasodilation could improve cognition by increasing circulation, which in turn leads to improved cerebral oxygenation in a similar manner to that observed with pulsed electromagnetic fields (Bragin et al., 2015).

## Conclusions

In conclusion, the results from the current study provide support to the hypothesis that the use of a fNIRS device may induce PBM-related processes and therefore have an effect on cognition (Martini & Arias, 2021). To our knowledge, this is the first study using a fNIRS device to induce PBM, and considering our limited sample size, further evidence is needed to confirm such a hypothesis. Nonetheless, the present results may have a significant impact on the way fNIRS are conceived and consequently serve as a wake-up call for the scientific community. All future neuroscientific studies using this technology may need to consider the modulatory effects intrinsic in the usage of fNIRS when assessing the efficacy/effects of a possible intervention on brain activation and related cognitive performance.

## Data Availability

All data generated or analyzed during this study are included in this published article.

## Code availability

All software are available online.
